# Paliperidone Reversion of Maternal Immune Activation-Induced Changes on Brain Serotonin and Kynurenine Pathways

**DOI:** 10.3389/fphar.2021.682602

**Published:** 2021-05-13

**Authors:** Karina S. MacDowell, Eva Munarriz-Cuezva, J. Javier Meana, Juan C. Leza, Jorge E. Ortega

**Affiliations:** ^1^ Department of Pharmacology and Toxicology, Faculty of Medicine, University Complutense of Madrid (UCM), Madrid, Spain; ^2^ Centro de Investigación Biomédica en Red de Salud Mental CIBERSAM, Bizkaia, Madrid, Spain; ^3^ Instituto de Investigación Hospital 12 de Octubre (i+12), IUIN-UCM, Madrid, Spain; ^4^ Department of Pharmacology, University of the Basque Country UPV/EHU, Leioa, Bizkaia, Spain; ^5^ Biocruces Bizkaia Health Research Institute, Barakaldo, Bizkaia, Spain

**Keywords:** schizophrenia, atypical antipsychotics, kynurenine, serotonin, maternal immune activation, polyinosinic:polycytidylic acid (poly(I:C))

## Abstract

Emerging evidence indicates that early-life exposure to environmental factors may increase the risk for schizophrenia via inflammatory mechanisms. Inflammation can alter the metabolism of tryptophan through the oxidative kynurenine pathway to compounds with neurotoxic and neuroprotective activity and compromise serotonin (5-HT) synthesis. Here we investigate the role of serotonergic and kynurenine pathways in the maternal immune activation (MIA) animal model of schizophrenia. The potential reversion exerted by long-term antipsychotic treatment was also evaluated. MIA was induced by prenatal administration of polyinosinic:polycytidylic acid (poly (I:C)) in mice. Expression of different proteins and the content of different metabolites involved in the function of serotonergic and kynurenine pathways was assessed by RT-PCR, immunoblot and ELISA analyses in frontal cortex of the offspring after puberty. MIA decreased tissue 5-HT content and promoted changes in the expression of serotonin transporter, 5-HT_2A_ and 5-HT_2C_ receptors. Expression of indoleamine 2,3-dioxygenase 2 (IDO2) and kynurenine 3-monooxygenase (KMO) was increased by poly (I:C) whereas kynurenine aminotransferase II and its metabolite kynurenic acid were not altered. Long-term paliperidone was able to counteract MIA-induced changes in 5-HT and KMO, and to increase tryptophan availability and tryptophan hydroxylase-2 expression in poly (I:C) mice but not in controls. MIA-induced increase of the cytotoxic risk ratio of kynurenine metabolites (quinolinic/kynurenic acid) was also reversed by paliperidone. MIA induces specific long-term brain effects on serotonergic activity. Such effects seem to be related with alternative activation of the kynurenine metabolic pathway towards a cytotoxic status. Atypical antipsychotic paliperodine partially remediates abnormalities observed after MIA.

## Introduction

Evidence accumulated over the past decades supports the neurodevelopmental origin of schizophrenia. Specific alterations in brain development induced by endogenous or exogenous factors that might be pivotal for the emergence of the disease have begun to be identified (Marenco and Weinberger, 2000; Lewis and Levitt, 2002; Rapoport et al., 2012; Schmidt and Mirnics, 2015). Schizophrenia symptoms are divided into three core clusters; positive (delusion, hallucinations, disturbances of thoughts and paranoia), negative (apathy, speech poverty, affect flattering, social withdrawal and anhedonia), and cognitive symptoms (attention and memory deficits and difficulties in planning and organizing life). Although onset is typically in late adolescence or early adulthood, certain clinical features of schizophrenia can be seen in an earlier prodromal phase (Lewis and Levitt, 2002). Signs of the different dysfunctions depend on affected neural substrates, as the frontal cortex (FC), and appear to emerge during the protracted maturation of the neural circuitries throughout childhood and adolescence (Hoftman et al., 2017).

Epidemiological studies have identified several environmental disturbances during critical developmental periods that confer increased risk of brain disorders (Tsuang, 2000; Sullivan et al., 2003; van Os et al., 2010). The fact that the increased risk of schizophrenia in offspring might be induced by a variety of bacteria-, virus-, and parasite-induced maternal infections during pregnancy suggests that immune-activating agents *per se* are not the critical risk factor and point to the importance of the maternal and fetal immune response in the future emergence of the disease (Brown, 2006; Patterson, 2007; Boksa, 2008; Khandaker et al., 2015). In that sense, numerous investigations have reported alterations in inflammatory/immune signalling and its regulatory mechanisms in the pathophysiology of schizophrenia when evaluated in peripheral blood cells and in *postmortem* brain studies (Khandaker et al., 2015; Leza et al., 2015). An imbalance between pro- and anti-inflammatory signalling has been observed in subjects with a first episode of psychosis (García-Bueno et al., 2014a; García-Bueno et al., 2014b) and in schizophrenia (Martínez-Gras et al., 2011). In the same line of evidence, different meta-analyses indicate increased levels of inflammatory cytokines in schizophrenia and psychosis (Miller et al., 2011; Upthegrove et al., 2014; Goldsmith et al., 2016; Fraguas et al., 2019). However, how these inflammatory and neuroinmmune alterations are able to alter the different neurotransmission systems implicated in schizophrenia remains unclear.

One of the neurotransmission systems broadly involved in schizophrenia is the serotonergic system. It has long been recognized that psychedelic drugs, such as lysergic acid diethylamide (LSD), psilocybin and mescaline, recruit specific serotonin 5-HT_2A_ receptor (5-HT_2A_R)-mediated signaling pathways to exert their psychotic-like effects (González-Maeso et al., 2007; Geyer and Vollenweider, 2008). Indeed, they are agonists/partial agonists at the 5-HT_2A_Rs inducing symptoms in healthy volunteers that resemble and share similarities with the core symptoms of schizophrenia (Gouzoulis-Mayfrank et al., 2005; Carhart-Harris et al., 2012). In addition, there is a good correlation between 5-HT_2A_R binding affinities and hallucinogenic potencies of psychoactive drugs such as LSD and N,N-dimethyltryptamine in humans (Glennon et al., 1984). These facts, together with the higher affinity of atypical antipsychotic agents such as risperidone/paliperidone, clozapine and olanzapine, among others, for 5-HT_2A_Rs as compared to dopamine D_2_ receptors, led to the serotonin hypothesis of schizophrenia with 5-HT_2A_Rs being recognized as key players (Geyer and Vollenweider, 2008; Gonzalez-Maeso and Sealfon, 2009). Nevertheless, studies carried out on *postmortem* human brain of subjects with schizophrenia show some inconsistencies in the variation of serotonin (5-HT) concentrations and 5-HT_2A_R densities, so it is not clear enough so far, whether an increase or a decrease in those levels occurs in the disease (Lovett Doust et al., 1975; Crow et al., 1979; Bleich et al., 1988; Iqbal et al., 1991; Ohuoha et al., 1993; Muguruza et al., 2013).

Therefore, to understand the mechanisms underlying schizophrenia, it is essential to focus on the long-term disturbances of postnatal brain maturation and to consider how the initial insults in early development affect this process. Animal models provide fundamental knowledge about the neurobiological mechanisms of human brain disorders. Based on construct premises described above, maternal immune activation (MIA) during pregnancy has become one of the most validated animal models to study schizophrenia-like symptoms. In these models, pregnant animals are exposed to immunological stimulation at a specific gestational stage. An extended *in-utero* immune activation is commonly modeled by injecting the viral-mimetic compound polyinosinic:polycytidylic acid (poly (I:C)) in pregnant dams. This viral-like administration results in activation of Toll-like receptor 3 (TLR3), a member of TLR family of pattern recognition receptors of the innate immune system, stimulating maternal production and release of many pro-inflammatory cytokines (Cunningham et al., 2007; Estes and McAllister, 2016). As consequence, the integrity of the placental barrier is altered, allowing the entrance of maternal-derived cytokines that would modify the physiological embryonic development and exert long-term detrimental effects in the offspring (Meyer, 2014; Estes and McAllister, 2016). Different behavioral and morphofunctional brain abnormalities in offspring of poly (I:C)-treated dams have been observed after puberty (Zuckerman et al., 2003; Ozawa et al., 2006; Meyer and Feldon, 2012; Meyer, 2014; Luchicchi et al., 2016). In concordance with findings in brain of subjects with schizophrenia, previous works confirmed that this MIA model promotes the accumulation of pro-inflammatory mediators such as different cytokines, as well as intracellular inflammatory and oxido/nitrosative mediators such as the transcription factor NFkB or the inducible nitric oxide synthase (iNOS) (Song et al., 2011; Volk et al., 2015; MacDowell et al., 2017; Goh et al., 2020). All these abnormalities suggest that MIA induced by poly (I:C) promotes in offspring a face-valid schizophrenia-like model. Regarding the 5-HT system, an altered serotonergic axonal circuit formation after MIA has been reported (Goeden et al., 2016).

The serotonergic and the inflammatory hypotheses of schizophrenia might be complementary. Aberrant deviation of tryptophan metabolism to alternative pathways is induced by the activity of the enzyme indoleamine 2,3-dioxygenase (IDO). Pro-inflammatory cytokines such as interleukin-2, interferon-γ, and tumor necrosis factor-α activate IDO, which promotes depletion of 5-HT and generates a range of metabolites involved in inflammation, immune response and excitatory neurotransmission (Hassanain et al., 1993; Stone et al., 2013; Cervenka et al., 2017). Among the different tryptophan-dependent metabolites synthesized by IDO, quinolinic acid (QUIN) and kynurenic acid (KYNA) have been implicated in the neurobiology of brain disorders (Cervenka et al., 2017). QUIN is associated with neural excitotoxicity through its N-methyl-D-aspartate receptor (NMDAR) agonist properties whereas KYNA exerts neuronal protection due to its ability to antagonize NMDARs (Stone, 2020). It has been proposed that an aberrant function of the kynurenine pathway could induce an imbalance between excitotoxicity/neuroprotection mechanisms that finally affect FC integrity (Stone et al., 2013). Different studies have proposed that beyond the acute mechanism of action of atypical antipsychotic drugs, long-term mechanisms include an important anti-inflammatory/antioxidant effect, leading to potential cytoprotective activity (Drzyzga et al., 2006; Sugino et al., 2009; Ribeiro et al., 2013; de Witte et al., 2014; MacDowell et al., 2016; MacDowell et al., 2017; Casquero-Veiga et al., 2019). In this regard, paliperidone administration in mice at equivalent dose to used in human therapeutics seems to block the impairments induced by MIA (MacDowell et al., 2017). However, the long-term consequences on tryptophan metabolic pathways that could underlay the efficacy of atypical antipsychotic treatment are, to date, not sufficiently explained.

The aim of the present study was to evaluate whether maternal viral-like immune activation in early/middle gestation in mice may induce long-lasting neurochemical changes in the serotonergic and kynurenine pathways in the FC of adult offspring. In addition, the effect of long-term atypical antipsychotic paliperidone treatment on the MIA-induced alterations was also evaluated.

## Material and Methods

### Animals and Experimental Model

Pregnant C57BL/6J mice (Envigo, Spain) were injected i.p. with either 5 mg/kg poly (I:C) (Sigma-Aldrich, United States) or the vehicle (saline solution) on gestational day 9.5. This period in mice corresponds to the second third period of pregnancy (Clancy et al., 2001) and represents a critical moment of brain development with events that in human gestation occur in the middle/end of the first trimester (Schepanski et al., 2018). Doses above 5 mg/kg administered on GD 9.5 have previously shown to produce physiological, behavioral and neurochemical changes in the offspring of these mice (Meyer et al., 2008; Holloway et al., 2013; Prades et al., 2017; MacDowell et al., 2017). Animals were maintained under standard temperature and humidity conditions in a 12 h light/dark cycle (lights on at 08:00 h) with free access to food and water. All experimental protocols were approved by the Animal Welfare Committee of the University of the Basque Country and adhered to the guidelines of the European legislation (European Union Directive 2010/63/UE).

### Drug Administration and Experimental Designs

Male and female pups were born from poly (I:C)-treated and saline-treated dams and were randomly assigned among four treatment groups with variables of pre-treatment (poly (I:C) *vs*. saline) and drug (paliperidone vs. vehicle). Paliperidone (PubChem CID:115237; Sigma-Aldrich, Spain) was dissolved in saline solution with 0.26 mM of acetic acid (vehicle, Veh) to obtain 0.01 mg/ml, pH adjusted to 7.4. Young-adult animals (≥60 postnatal days; PND) originating from the different litters were injected i.p. with either paliperidone (0.05 mg/kg, 5 ml/kg) or vehicle (5 ml/kg) for 21 consecutive days. Group sample sizes (sex balanced) were poly (I:C)/vehicle, *n* = 9; poly (I:C)/paliperidone, *n* = 8; saline/vehicle, *n* = 11; saline/paliperidone, *n* = 8. The dose and duration of paliperidone treatment was selected to be similar to commonly prescribed human dosages for a 50 kg adolescent subject, and on the basis of previous *in vivo* determinations of behavioral and brain structural abnormalities in poly (I:C) offspring during adulthood (Piontkewitz et al., 2011; Richtand et al., 2011; MacDowell et al., 2017). No differences in body weight between the four animal groups throughout the study were observed (data not shown).

### Preparation of Biological Samples

The animals were subjected to cervical dislocation 48 h after of the last dose of paliperidone or vehicle treatments. This washout period is considered adequate to overcome acute effects due to residual presence of the drug and focus the findings on modulation exerted by chronic paliperidone administration (MacDowell et al., 2017). The brain was removed from the skull and, after careful removal of the meninges and blood vessels, frontal cortical areas from both brain hemispheres were excised and frozen at −80°C until assayed.

### Western Blot Analysis

Brain FC samples were homogenized by sonication in PBS (pH = 7) mixed with a protease inhibitor cocktail (Complete^®^, Roche, Spain). After determining and adjusting protein levels, homogenates of FC tissue were mixed with Laemmli sample buffer (Bio-Rad, United States) and β-mercaptoethanol (50 μL/ml Laemmli), and 15 µg protein were loaded into an electrophoresis gel. Once separated on the basis of molecular weight, proteins from the gels were blotted onto a nitrocellulose membrane with a semi-dry transfer system (Bio-Rad, United States) and were incubated with specific antibodies against: 1) tryptophan hydroxylase 2 (TPH2, 1:1000 in BSA 1%; ab184505, abcam, United Kingdom); 2) serotonin transporter (SERT, 1:750 in BSA 2,5%; sc1458, Santa Cruz, United States); 3) serotonin 5-HT_2A_ receptor (5-HT_2A_R, 1:1000; sc50397, Santa Cruz, United States); 4) serotonin 5-HT_2C_ receptor (5-HT_2C_R, 1:1000; sc17797, Santa Cruz, United States); 5) monoamine oxidase A (MAO-A, 1:1000; ab126751, abcam, United Kingdom); 6) indoleamine 2,3-dioxygenase 1 (IDO1, 1:750 in BSA 1%; sc25809, Santa Cruz, United States); 7) indoleamine 2,3-dioxygenase 2 (IDO2, 1:750 in BSA 1%; NBP2-44174, Novusbio, United Kingdom); 8) kynurenine 3-monooxygenase (KMO, 1:750 in BSA 1%; NBP2-29936, Novusbio, United Kingdom); 9) kynurenine aminotransferase 2 (KATII, 1:1000; sc377158, Santa Cruz, United States) and 10) β-actin (1:10,000; A5441, Sigma-Aldrich, United States). Primary antibodies were recognized by the respective horseradish peroxidase-linked secondary antibodies. Blots were imaged using an Odyssey Fc System (Li-COR, Biosciences, Germany) and were quantified by densitometry (NIH ImageJ software). In all the western-blot analyses, the housekeeping β-actin was used as loading control for normalization. One sample was repeated in every blot as a variability control to check the possible interassay unevenness, and each sample was analyzed at least three times in separate assays. The mean value of each animal were treated as a single measurement for data analyses. The data are presented as percentage change respect to the control group.

### Kynurenine Pathway Metabolites and Serotonin Measurement

The levels of tryptophan (BAE-2700, ImmuSmol, United States), kynurenine (BAE-2200, ImmuSmol, United States), kynurenic acid (KYNA, CED718Ge, Cloud-Clone, United States), quinolinic acid (QUIN; CEK552Ge, Cloud-Clone, United States) and 5-HT (BAE-5900, LDN, Germany) in brain FC homogenate samples were detected using a commercially available ELISA-based kit following the manufacturer’s instructions. The intra- and interassay coefficient of variation were 11 and 8.4% for tryptophan, 10.3 and 13.3% for kynurenine, 10 and 12% for KYNA and QUIN, 4.1and 9.4% for 5-HT, respectively.

### Quantitative Real-Time Polymerase Chain Reaction Assays

mRNA expression of serotonin 5-HT_1A_, 5-HT_1B_, 5-HT_2A_, 5-HT_2B_, 5-HT_2C_ and 5-HT_7_ receptors, KAT I, II and III, KMO, glyceraldehyde-3-phosphate dehydrogenase (GADPH) and tubulin was evaluated as described in Supplementary information.

### Statistical Analyses

The ROUT method was performed with a significance Q set at 1% for the detection of outliers. Two-way ANOVA was used for comparisons, considering as the first factor the presence or absence of poly (I:C) and as second factor the presence or absence of paliperidone treatment. Bonferroni *post hoc* test was applied in case of significant interaction (F value *p* < 0.05) between factors. All the results of the ANOVA analyses (*F* values and *dfs*) are included in Table 1. A *p* value ≤0.05 was defined as statistically significant. The data were analysed using GraphPad Prism. The data and figures are expressed as mean ± SEM.

**TABLE 1:** Two-way ANOVA analyses (F, df and *p* values) of protein expression and metabolites determination. Bold: statistically significant values. WB, western blot experiments.

Parameter	Poly (I:C)	Treatment	Interaction
Tryptophan	F_(1,29)_ = 0.03; *p* = 0.872	**F**_**(1,29)**_ **= 6.73; *p* = 0.014**	**F**_**(1,29)**_ **= 4.32; *p* = 0.046**
WB TPH2	**F**_**(1,29)**_ **= 8.04; *p* = 0.008**	F_(1,29)_ = 2.33; *p* = 0.137	**F**_**(1,29)**_ **= 6.23; *p* = 0.018**
5-HT	**F**_**(1,28)**_ **= 5.48; *p* = 0.026**	**F**_**(1,28)**_ **= 5.67; *p* = 0.024**	F_(1,28)_ = 0.08; *p* = 0.773
WB SERT	**F**_**(1,29)**_ **= 18.22; *p* < 0.001**	F_(1,29)_ = 2.52; *p* = 0.123	F_(1,29)_ = 0.72; *p* = 0.404
WB MAO-A	F_(1,29)_ = 0.18; *p* = 0.671	F_(1,29)_ = 0.31; *p* = 0.581	F_(1,29)_ = 0.02; *p* = 0.902
WB 5-HT_2A_R	**F**_**(1,29)**_ **= 6.97; *p* = 0.013**	F_(1, 29)_ = 0.13; *p* = 0.721	F _(1, 29)_ = 0.03; *p* = 0.872
WB 5-HT_2C_R	**F**_**(1,29)**_ **= 7.97; *p* = 0.008**	F_(1,29)_ = 0.25; *p* = 0.623	F_(1,29)_ = 0.02; *p* = 0.895
WB IDO1	F_(1,29)_ = 0.00; *p* = 0.984	**F**_**(1,29)**_ **= 12.17; *p* = 0.001**	**F**_**(1,29)**_ **= 8.32; *p* = 0.007**
WB IDO2	**F**_**(1,29)**_ **= 8.53; *p* = 0.006**	F_(1,29)_ = 1.45; *p* = 0.238	F_(1,29)_ = 0.52; *p* = 0.477
Kynurenine	**F**_**(1,29)**_ **= 5.68; *p* = 0.023**	F_(1,29)_ = 0.22; *p* = 0.645	F_(1,29)_ = 0.01; *p* = 0.907
WB KMO	**F**_**(1,29)**_ **= 5.28; *p* = 0.003**	**F**_**(1,29)**_ **= 10.99; *p* = 0.002**	**F**_**(1,29)**_ **= 8.41; *p* = 0.007**
QUIN	F_(1,29)_ = 3.37; *p* = 0.076	F_(1,29)_ = 0.34; *p* = 0.566	F_(1,29)_ = 0.27; *p* = 0.606
WB KATII	F_(1,29)_ = 2.73; *p* = 0.109	F_(1,29)_ = 0.14; *p* = 0.706	F_(1,29)_ = 0.02; *p* = 0.880
KYNA	F_(1,29)_ = 1.34; *p* = 0.256	F_(1,29)_ = 0.32; *p* = 0.574	F_(1,29)_ = 0.08; *p* = 0.772
QUIN/KYNA ratio	F_(1,29)_ = 2.85; *p* = 0.102	F_(1,29)_ = 3.61; *p* = 0.067	**F**_**(1,29)**_ **= 6.99; *p* = 0.013**

## Results

### Effects of Paliperidone on Serotonin Neurotransmission After Maternal Immune Activation

**FIGURE 1:**
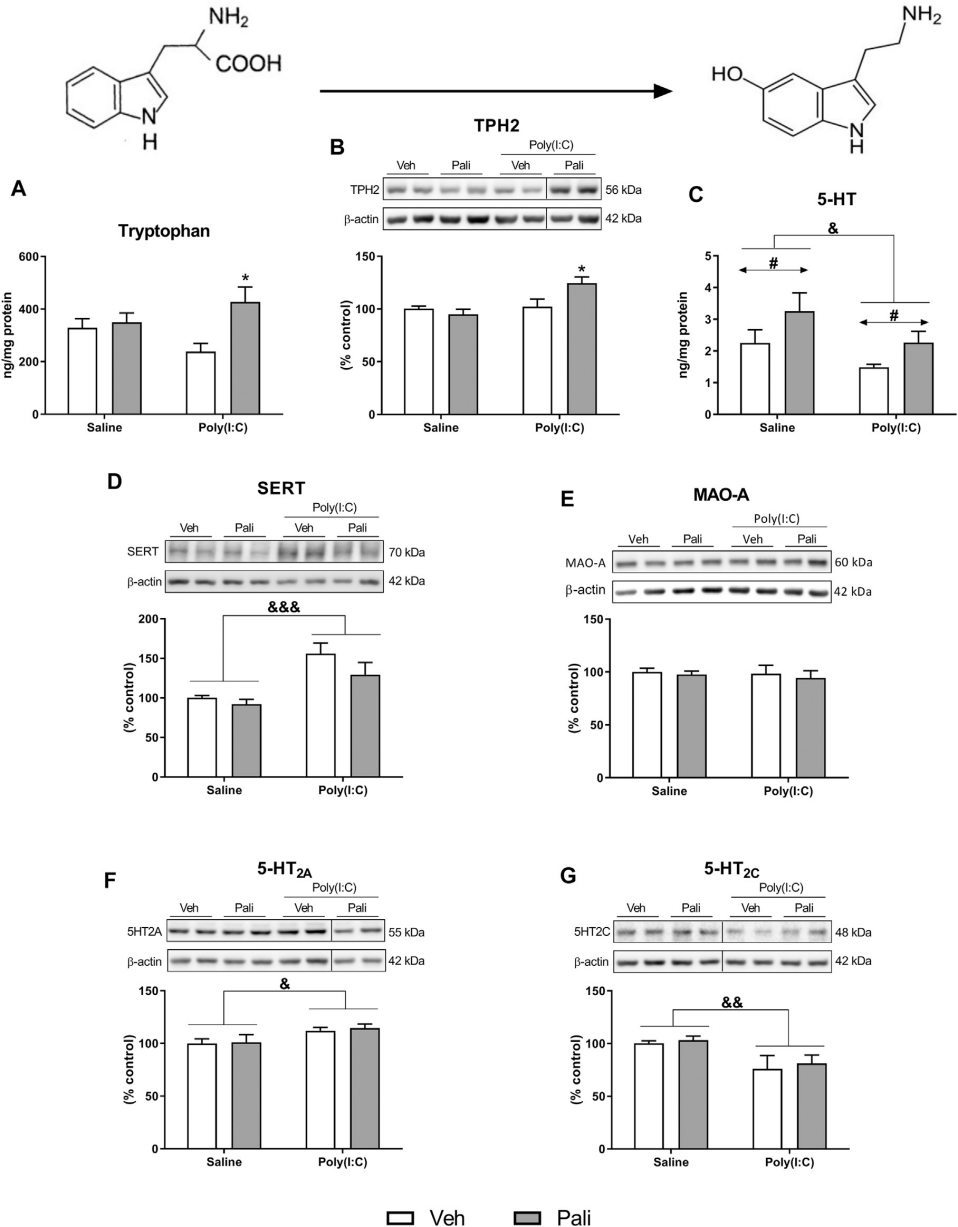
Effects of paliperidone on 5-HT neurochemical processes after MIA in mice FC. Tryptophan levels **(A)**, protein expression of TPH2 **(B)**, 5-HT levels **(C)** and protein expression of SERT, MAO-A, 5-HT_2A_ and 5-HT_2C_ receptors **(D–G)** in the FC of mice treated with vehicle (Veh) or paliperidone (Pali) in control (Saline) and prenatal poly (I:C) administration. The densitometric data of the respective immunoreactive bands were normalized by β-actin. In the **(B,F,G)** panels, blots were cropped (black lines) for improving the clarity and conciseness of the presentation. Bars represent means ± SEM. The number of experiments in all parameters analyzed was between 8-9 animals. Two-way ANOVA: ^&^ *p* <0.05, ^&&^ *p* < 0.01, ^&&&^ *p* < 0.001 for poly(I:C) factor; ^#^ *p* < 0.05 for treatment factor. *p < 0.05 vs poly (I:C)+Veh group (Bonferroni *post hoc* test after significant interaction).

Two-way ANOVA analysis of tryptophan levels in brain FC samples showed a significant effect for paliperidone treatment (F_(1,29)_ = 6.73, *p* = 0.014) and interaction between factors (F_(1,29)_ = 4.32, *p* = 0.046). Bonferroni *post hoc* test demonstrated that the increase in tryptophan levels affected to animals exposed to MIA treated with paliperidone (Figure 1A).

The conversion from tryptophan to 5-HT depends on the enzyme TPH2. A significant effect of poly (I:C) exposure (F_(1,29)_ = 8.04, *p* = 0.008) and interaction between main factors (F_(1,29)_ = 6.23, *p* = 0.018) were found. *Post hoc* analyses showed increased protein expression of TPH2 in animals exposed to poly (I:C) that were treated with paliperidone compared to the poly (I:C) group treated with vehicle (*p* < 0.05) (Figure 1B). In contrast, *post hoc* analyses did not revealed differences between saline and poly (I:C) groups in absence of paliperidone treatment. In addition, animals exposed to MIA showed a significant decrease of brain FC 5-HT levels, and paliperidone treatment induced an increment of this neurotransmitter in both control and poly (I:C) conditions (F_(1,28)_ = 5.48, *p* = 0.026 and F_(1,28)_ = 5.67, *p* = 0.024, respectively) (Figure 1C).

Other critical elements in the activity of the serotonergic system were analysed. Two-way ANOVA analysis revealed that animals exposed to MIA showed a significant increase in the protein expression of SERT (F_(1,29)_ = 18.22, *p* = 0.0002) and 5-HT_2A_Rs (F_(1,29)_ = 6.97, *p* = 0.013) together with a decrease in the protein expression of 5-HT_2C_Rs (F_(1,29)_ = 7.97, *p* = 0.008) (Figures 1D,F,G). 5-HT_2A_R/5-HT_2C_R protein ratio was increased after MIA insult (Supplementary Figure S1). However, no differences were found between groups in the protein expression of MAO-A (Figure 1E). At this point, paliperidone did not exert any influence on the FC protein expression of these elements of serotonergic neurotransmission system. In addition, different 5-HT receptors were also evaluated at mRNA expression level. The only observed alterations of mRNA expression were increased 5-HT_2A_Rs linked to poly (I:C) and decreased 5-HT_2C_Rs induced by paliperidone in poly (I:C) group (Supplementary Figure S2 and Table S2).

### Effects of Paliperidone on Kynurenine Pathway After Maternal Immune Activation

**FIGURE 2:**
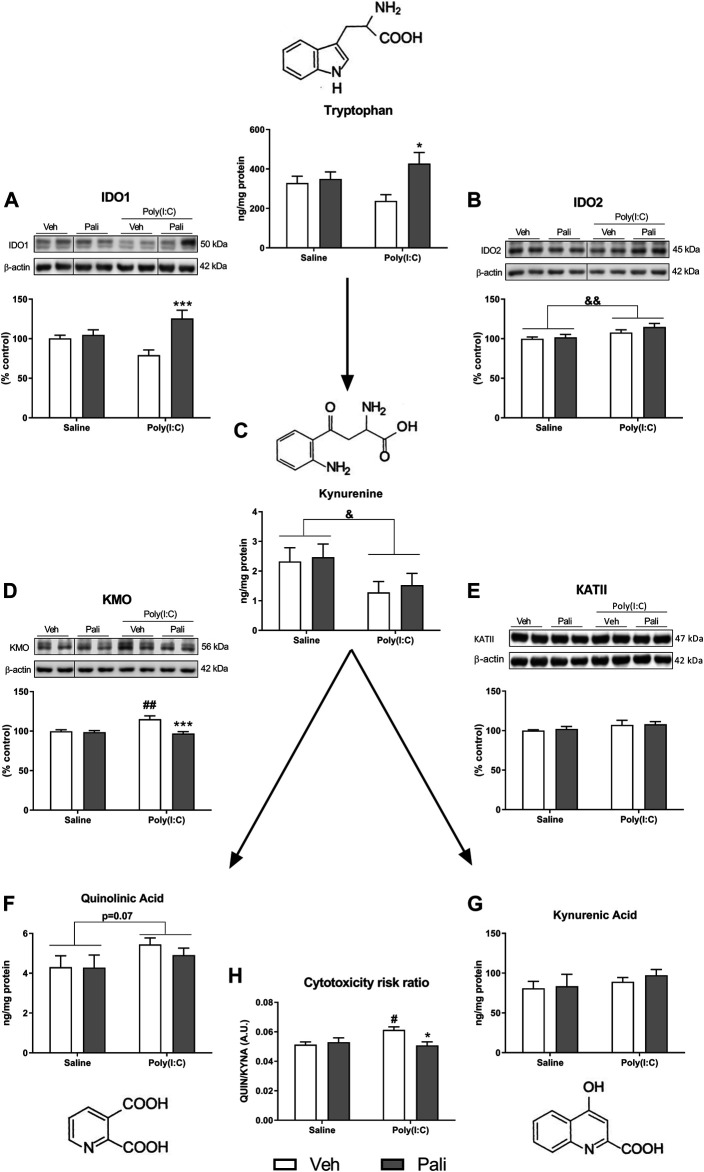
Effects of paliperidone on kynurenine metabolic pathways after MIA in mice FC. Protein expression of IDO1 and IDO2 **(A,B)**, kynurenine levels **(C)**, protein expression of KMO and KATII **(D,E)**, QUIN levels **(F)**, KYNA levels **(G)** and cytotoxicity risk ratio **(H)** in the FC of mice treated with vehicle (Veh) or paliperidone (Pali) in control (Saline) and prenatal poly (I:C) administration. The densitometric data of the respective immunoreactive bands were normalized by β-actin. In the **(A,B,D)** panels, blots were cropped (black lines) for improving the clarity and conciseness of the presentation. Bars represent means ± SEM. The number of experiments in all parameters analyzed was between 8-9 animals.Two-way ANOVA: ^&^ *p* <0.05 and ^&&^ *p* <0.01 for poly(I:C) factor. ^#^ *p* <0.05, ^##^ *p* <0.01 vs saline+Veh group; *p < 0.05, ***p < 0.001 vs poly (I:C)+Veh group (Bonferroni *post hoc* test after significant interaction).

Tryptophan is also metabolized to kynurenine in the brain by the enzymes IDO1 and IDO2. Two-way ANOVA analysis of the IDO1 protein expression reported a significant effect of paliperidone treatment and interaction (F_(1,29)_ = 12.17, *p* = 0.001 and F_(1,29)_ = 8.32, *p* = 0.007 respectively). *Post hoc* analyses showed that paliperidone treatment increased IDO1 protein expression in MIA-exposed (*p* < 0.001; Figure 2A) but not in saline-treated animals. In addition, the analysis for IDO2 revealed a significant stimulatory effect of poly (I:C) (F_(1,29)_ = 8.53, *p* = 0.006; Figure 2B). Furthermore, the two-way ANOVA analysis also indicated a significant decreasing effect of MIA exposure on kynurenine levels (F_(1,29)_ = 5.68, *p* = 0.023; Figure 2C).

Kynurenine can be transformed into two metabolites with opposite effects. First, kynurenine is metabolized to QUIN through multiple-step reactions, that involve KMO enzyme. Second, kynurenine can be transformed into KYNA mainly by the KATII enzyme. The mRNA expression of KMO and KAT isoforms was evaluated. KMO expression increased but not the different KAT isoforms in response to MIA (Supplementary Figure S1 and Table S2). When protein expression levels were analysed, two-way ANOVA analysis of KMO found a significant effect of MIA, paliperidone treatment and interaction (F_(1,29)_ = 5.28, *p* = 0.003; F_(1,29)_ = 10.99, *p* = 0.002; and F_(1,29)_ = 8.41, *p* = 0.007, respectively). *Post hoc* analyses reported that MIA increased KMO expression (*p* < 0.01) and that paliperidone treatment decreased KMO protein expression in MIA conditions (*p* < 0.001) (Figure 2D). In contrast, no significant change was found in the KATII protein expression analyses (Figure 2E). Regarding the kynurenine metabolites, a trend to increase of QUIN levels by MIA exposure was observed, although it did not reach statistical significance (F_(1,29)_ = 3.37; *p* = 0.076) (Figure 2F). However, for KYNA levels, no alterations were observed (Figure 2G).

Finally, the ratio between QUIN and KYNA levels was calculated as a possible indicator of cytotoxicity risk. Two-way ANOVA analysis indicated a significant effect of interaction between the factors (F_(1,29)_ = 6.99; *p* = 0.013). *Post hoc* analyses reported that MIA animals showed an increased cytotoxic ratio compared to saline group in absence of treatment (*p* < 0.05) and that long-term paliperidone is able to reverse such increase in poly (I:C) MIA animals (*p* < 0.05) (Figure 2H).

## Discussion

The present study shows that a single maternal exposure to the immunoreactive agent poly (I:C) in early/middle brain development during pregnancy in mice is sufficient to induce long-lasting changes of the 5-HT neurotransmission system in the FC of adult offspring. This fact seems to match with the activation of alternative metabolic pathways of L-tryptophan, probably induced by the presence of pro-inflammatory conditions in the brain of affected mice (Song et al., 2011; Volk et al., 2015; MacDowell et al., 2017). Indeed, an imbalance between metabolic products of the kynurenine pathway emerges shifting towards a pro-cytotoxic status. Previous studies performed in the MIA animal model generated in the same mice strain (C57BL/6), at the same dose and route of administration of poly (I:C) (5 mg/kg i.p.) and at the same gestational day (9.5) have shown behavioral abnormalities (MacDowell et al., 2017; Prades et al., 2017). These facts support the idea that MIA-induced impacts in serotonergic and kynurenine pathways may contribute to the behavioral impairments in the model. On the other hand, chronic paliperidone treatment counterbalances the impairments induced by MIA insult and thus, it could partially explain the ability of atypical antipsychotics to ameliorate some of the schizophrenia-related behavioral impairments previously observed in this specific animal model of the disorder (Meyer et al., 2010; Piontkewitz et al., 2011; Richtand et al., 2011; Dickerson and Bilkey, 2013; MacDowell et al., 2017).

### Effects of Paliperidone on Serotonin Neurotransmission After Maternal Immune Activation

In mice subjected to MIA, a decrease on tissue 5-HT content was observed. The finding agrees with the observation that intracerebroventricular injection of the cytokines that are released in response to poly (I:C) (Meyer et al., 2006; Cunningham et al., 2007) decreases 5-HT concentrations in the FC (Kamata et al., 2000). Likewise, a decrease in the rat brain cortical 5-HT content was observed in the offspring born from bacterial toxin lipopolysaccharide-immune-challenged mothers (Swanepoel et al., 2018). However, there are also studies reporting unaltered tissue 5-HT concentrations in the FC of MIA animal models (Winter et al., 2009; Abazyan et al., 2010; Hadar et al., 2015; Goh et al., 2020). The discrepancies highlight the importance of the intensity of MIA, as evidenced by the dose of immunogenic substance used, as well as the impact on the neurodevelopmental trajectory in relation to gestation time for the MIA. The 5-HT decrease was reversed by paliperidone treatment, demonstrating that antipsychotic drugs can ameliorate the consequences of prenatal MIA insult on neurotransmitter concentrations. In agreement with the results presented here, a role for 5-HT alterations in the pathophysiology of schizophrenia has been repeatedly postulated. It has been proposed that 5-HT dysregulation in schizophrenia patients could be the cause of the negative symptoms and high rates of comorbid depression observed in the disorder (Abi-Dargham, 2007; Selvaraj et al., 2014). In that sense, reduction of brain 5-HT synthesis exerted by acute tryptophan depletion (Carpenter et al., 1998), has been shown to worsen negative symptoms (Sharma et al., 1997). In addition, selective serotonin reuptake inhibitors have been proven to be effective in treatment of the negative symptoms of the disease (Singh et al., 2010). Monoamine tissue concentrations are mainly the end result of synthesis from amino acid precursors and reuptake mechanism from the extracellular space. In the present study, tissue levels of the 5-HT precursor L-tryptophan and the TPH2, considered the limiting synthesis enzyme, were not significantly altered by MIA under basal conditions. Interestingly, paliperidone treatment increased the availability of the precursor L-tryptophan and TPH2 protein expression in poly (I:C) mice but not in controls, suggesting that long-term antipsychotic treatment could trigger compensatory mechanisms that contribute to restoration of the tissular content of 5-HT in the MIA model. However, tissue 5-HT content is also sensitive to paliperidone in control animals, which indicates the existence of other complementary mechanisms of paliperidone to increase tissue 5-HT content. The results seem to indicate that deficits of 5-HT in the cortex of poly (I:C) mice are not related to neurotransmitter synthesis alterations and point to a potential deviation of tryptophan metabolism towards alternative pathways, probably induced by the existence of a proinflammatory status in the FC. Regarding the other critical step in steady-state tissue 5-HT content, an increased expression of the selective reuptake protein SERT was observed in poly (I:C) MIA mice. Since this SERT overexpression did not overcome 5-HT depletion, it could be interpreted as a compensatory mechanism to restore adequate neurotransmitter levels more than a direct effect of MIA activation. In fact, no changes in SERT levels in frontal (Malkova et al., 2014), orbitofrontal and prelimbic cortex (Goh et al., 2020) have been seen in other studies of MIA poly (I:C) rodents. In contrast, another study revealed an increase of hippocampal SERT protein in poly (I:C) animals (20 mg/kg on gestational day 12.5) (Reisinger et al., 2016) suggesting extensive adaptation after MIA in different brain areas. In the present study, the influence of MIA on SERT was less apparent after paliperidone treatment, a condition in which 5-HT was higher, supporting to the hypothesis that SERT modulation represents a compensatory mechanism to overcome tissue 5-HT deficits. On the contrary, MAO-A, the catabolic enzyme that metabolizes different monoamines, including 5-HT, was not altered by either MIA or paliperidone treatment, ruling out the contribution of 5-HT metabolism to neurobiological alterations induced by the present MIA model.

In order to evaluate the MIA-induced alterations on 5-HT_2A_Rs and 5-HT_2C_Rs in the offspring, both the mRNA and the protein expression were evaluated. 5-HT_2A_R mRNA expression showed an increase in poly (I:C) mice that was associated to changes in protein expression. Similar findings have been previously reported in mice (Malkova et al., 2014) and rat offspring exposed prenatally to another immunoactive trigger (lipopolysaccharide on gestational days 15 and 16) (Wischhof et al., 2015). Radioligand binding assays also showed enhanced 5-HT_2A_R density in FC membranes of mice born from poly (I:C) and influenza-injected mothers (Moreno et al., 2011; Holloway et al., 2013). Moreover, it has been demonstrated that poly (I:C) offprings do not show basal hallucinogenic-like alterations but display supersensitivity of the head-twich response, a proxy of hallucinogenic behavior, to the administration of 5-HT_2A_R agonists such as 2,5-dimethoxy-4-iodoamphetamine (DOI) and LSD (Gonzalez-Maeso et al., 2007; Holloway et al., 2013; Malkova et al., 2014; Wischhof et al., 2015). This finding is compatible with a supersensitivity of 5-HT_2A_Rs. In the present study, MIA mice also showed reduced protein (but not mRNA) expression of 5HT_2C_Rs. This decrease could be a consequence of compensatory mechanisms induced by the 5-HT_2A_R dysfunction. Several lines of evidence demonstrated that 5-HT_2A_Rs and 5-HT_2C_Rs oppositely affect neurochemical release within the FC (Alex and Pehek, 2007) as well as inversely control of certain behaviors (Bubar and Cunningham, 2006). According to this, the present results also suggest the existence of a misbalance between 5-HT_2_R subtypes after MIA insult that could also contribute to alterations of serotonergic neurotransmission system. Paliperidone when administered at active therapeutic dose did not evoke any noteworthy effects on 5-HT_2A_R and 5-HT_2C_R protein expression in control and MIA animals. This seems to indicate that paliperidone reversion of impairments induced on the 5-HT system (present results) and behavior (MacDowell et al., 2017) by MIA are not the consequence of a direct modulation of the receptor expression by this antipsychotic drug. On the other hand, the relationships between MIA and the expression of the different 5-HT receptors is unknown. For this reason, complementary mRNA expression of 5-HT_1A_, 5-HT_1B_, 5-HT_2B_ and 5-HT_7_ receptors was performed. No differences were observed in the mRNA expression levels of these receptors neither in MIA mice nor in treated animals confirming that MIA seems to exert a selective alteration on 5-HT_2_Rs.

### Effects of Paliperidone on Kynurenine Pathway After Maternal Immune Activation

Kynurenine synthesis is one of the best-known alternative pathways of tryptophan metabolism. Tryptophan is deviated from 5-HT synthesis process to kynurenine pathway in response to inflammatory mediators acting on IDO enzymes. The kynurenine pathway yields different metabolites with neurotoxic and neuroprotective properties, the predominant action being dependent upon enzymes expression and cell-type activity (Stone and Darlington, 2002; Haroon et al., 2012; Cervenka et al., 2017). KYNA is a kynurenine metabolite synthesized mainly by KAT enzymes in astrocytes and that shows antagonist properties on NMDARs and α_7_-cholinergic nicotinic receptors. Another kynurenine metabolic product is QUIN, an agonist of the NMDARs (Stone and Darlington, 2002) that is mainly synthetized in microglia by KMO enzyme. Several lines of evidence indicate the existence of alterations in the main enzymes and metabolites of the kynurenine pathway after different MIA protocols (Khalil et al., 2013; Zavitsanou et al., 2014; Clark et al., 2019). These studies showed in rats that modulation is greatly influenced by the immunoactive dose, the gestational day of administration, and more importantly, by the postnatal age chosen for evaluation of the alterations (Clark et al., 2019). Accordingly, the present results showed a dysregulation of kynurenine pathway in adult mice littermates of poly (I:C) immuno-challenged mothers. Immunoblotting assays of IDO enzymes revealed that MIA offspring have increased protein expression of IDO2 but not IDO1 and that paliperidone treatment triggered a striking stimulatory effect on IDO1 protein expression, but did not influence IDO2. IDO1 represents a highly efficient for tryptophan metabolism under physiological conditions that is inducible by several pro-inflamatory factors and stress. In contrast, IDO2 physiological involvement is less known but transcripts are expressed in brain microglia (Brooks et al., 2017) and the enzyme seems to play a role under autoinmune inflammatory conditions (Larkin et al., 2016; Jusof et al., 2017). In the same line of evidence, KMO expression was increased by MIA and reversed after paliperidone treatment, whereas KATII remained unaffected. Probably, as consequence of these dysregulations, the intermediate metabolite kynurenine decreased in MIA mice. The finding suggests the existence of microglia activation as cellular substratum of KMO overexpression whereas astrocytes, the principal source of KATII, remain unaltered in response to prenatal administration of poly (I:C). More interestingly, QUIN but not KYNA, the end active products, seems to be increased in MIA mice, pointing to a selective hyperactivation of the cytotoxic arm of kynurenine pathway. KMO enzyme, similar to IDO, is induced by inflammatory mediators in several cell types whereas KAT isoforms are unimpacted (Fujigaki et al., 2017).

A fine-tuning of KYNA and QUIN levels seems to be a relevant factor in establishing a correct balance between the protective and deleterious effects induced respectively by these compounds through interaction with NMDARs. In the present study, the QUIN/KYNA ratio calculated as a cytotoxicity risk index increased in MIA groups and was reduced by long-term paliperidone treatment in the group of animals with prenatal MIA. These findings stress the importance of measuring the ratio of the different kynurenines more than particular metabolites as complementary indicators of kynurenine pathway imbalances in animal and human studies of neuropsychiatric disorders. In addition, it has been observed a decrease in the serum kynurenic acid concentration and an increase of quinolinic acid in rat littermates of poly (I:C) immuno-challenged mothers. This fact suggests that other tissues different than brain could be also affected by increased cytotoxicity risk index in MIA animals (Zavitsanou et al., 2014).

## Conclusion

The present study was prompted by previous findings demonstrating that the administration of paliperidone to offspring of poly (I:C)-treated dams prevented the emergence of proinflammatory and behavioral abnormalities in adulthood (MacDowell et al., 2017). According with the present results, the impairment of serotonergic function may be part of complex neuropathological processes induced by aberrant immunological activation of kynurenine pathways that contribute to a cytotoxic imbalance in the FC of poly (I:C) offspring mice, and that chronic treatment with paliperidone during adulthood may, at least in part, prevent such neuropathological processes. Further studies on the effects of infectious agents on neurodevelopment are required, particularly in view of recent outbreaks of infectious diseases (such as H1N1 influenza, Zika virus or more recently, SARS-CoV-2) suggesting that offspring of infected pregnant women might be high-risk groups for severe and long-lasting complications of psychiatric diseases.

## Data Availability

The raw data supporting the conclusions of this article will be made available by the authors, without undue reservation.

## Abbreviations

FC, frontal cortex; GADPH, glyceraldehyde-3-phosphate dehydrogenase; 5-HT, serotonin; 5-HT2AR, serotonin 5-HT2A receptor; 5-HT2CR, serotonin; 5-HT2C receptor; IDO, indoleamine 2,3-dioxygenase; iNOS, nitric oxide synthase; KAT, kynurenine aminotransferase; KMO, kynurenine 3-monooxygenase; KYNA, kynurenic acid; LSD, lysergic acid diethylamide; MAO-A, Monoamine oxidase-A; MIA, maternal immune activation; NFkB, nuclear factor kappa-light-chain-enhancer of activated B cells; NMDAR, N-methyl-D-aspartate receptor; Poly(I:C), polyinosinic:polycytidylic acid; QUIN, quinolinic acid; SERT, serotonin transporter; TLR3, toll-like receptor 3; TPH2, tryptophan hydroxylase-2.
