# Supplementary material for: Paliperidone Reversion of Maternal Immune Activation-Induced Changes on Brain Serotonin and Kynurenine Pathways
Source: Front Pharmacol. 2021 May 13;12:682602. doi: 10.3389/fphar.2021.682602 (PMC8156415; doi:10.3389/fphar.2021.682602)
Supplement: Supplementary file 1 [file DataSheet1.docx]

Supplementary Material

# Real Time-Polimerase Chain Reaction assays

Total cytoplasmic RNA was prepared from samples of brain FC using TRIZOL reagent (Invitrogen, New York, USA); aliquots were converted to complementary DNA using random hexamer primers. Quantitative changes in mRNA levels were estimated by real time-polymerase chain reaction using the following cycling conditions: 35 cycles of denaturation at 95°C for 10 s, annealing at 58–61°C for 15 s, depending on the specific set of primers for serotonin 5-HT_1A_, 5-HT_1B_, 5-HT_2A_, 5-HT_2B_, 5-HT_2C_ and 5-HT_7_ receptors, KATI, KATII, KATIII, KMO, glyceraldehyde-3-phosphate dehydrogenase (GADPH) and tubulin (see Table S1) and extension at 72°C for 20 s. Reactions were carried out in the presence of SYBR Green (1:10000 dilution of stock solution, Invitrogen, Spain), carried out in a 20 µl reaction in a Corbett Rotor-Gene (Corbett Research, Australia). Relative mRNA concentrations were calculated from the take-off point of reactions using the included software. GADPH and tubulin expression levels were used as housekeeping values to normalize data. No differences were found in GADPH and tubulin levels between all groups studied.

Primers were designed using Primer-BLAST (NCBI, USA), verified by a simulation in-silico and checked through blast (NCBI, USA). Triplicate measurements of each cDNA aliquot were performed and a negative internal control (without cDNA) was also included in each assay.

# Supplementary Figures and Tables

## Supplementary Figures


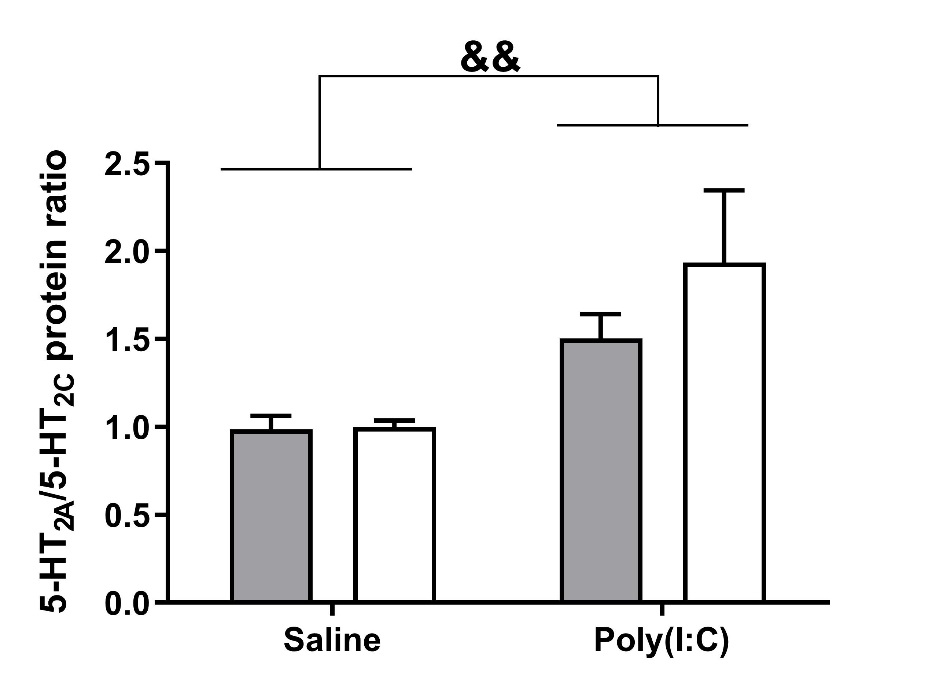


**Supplementary Figure 1.** Effects of paliperidone on 5-HT_2A_ and 5-HT_2C_ protein ratio after MIA in mice FC. Bars represent means±SEM. The number of experiments in all parameters analyzed was between 8-9 animals. Two-way ANOVA followed by Bonferroni post-hoc test. Poly(I:C): **F_(1,29)_=9.29; p<0.01**; treatment F_(1, 29)_=0.86; p=0.361; interaction: F _(1, 29)_=0.76; p=0.388). ^&&^p<0.01 for poly(I:C) factor.


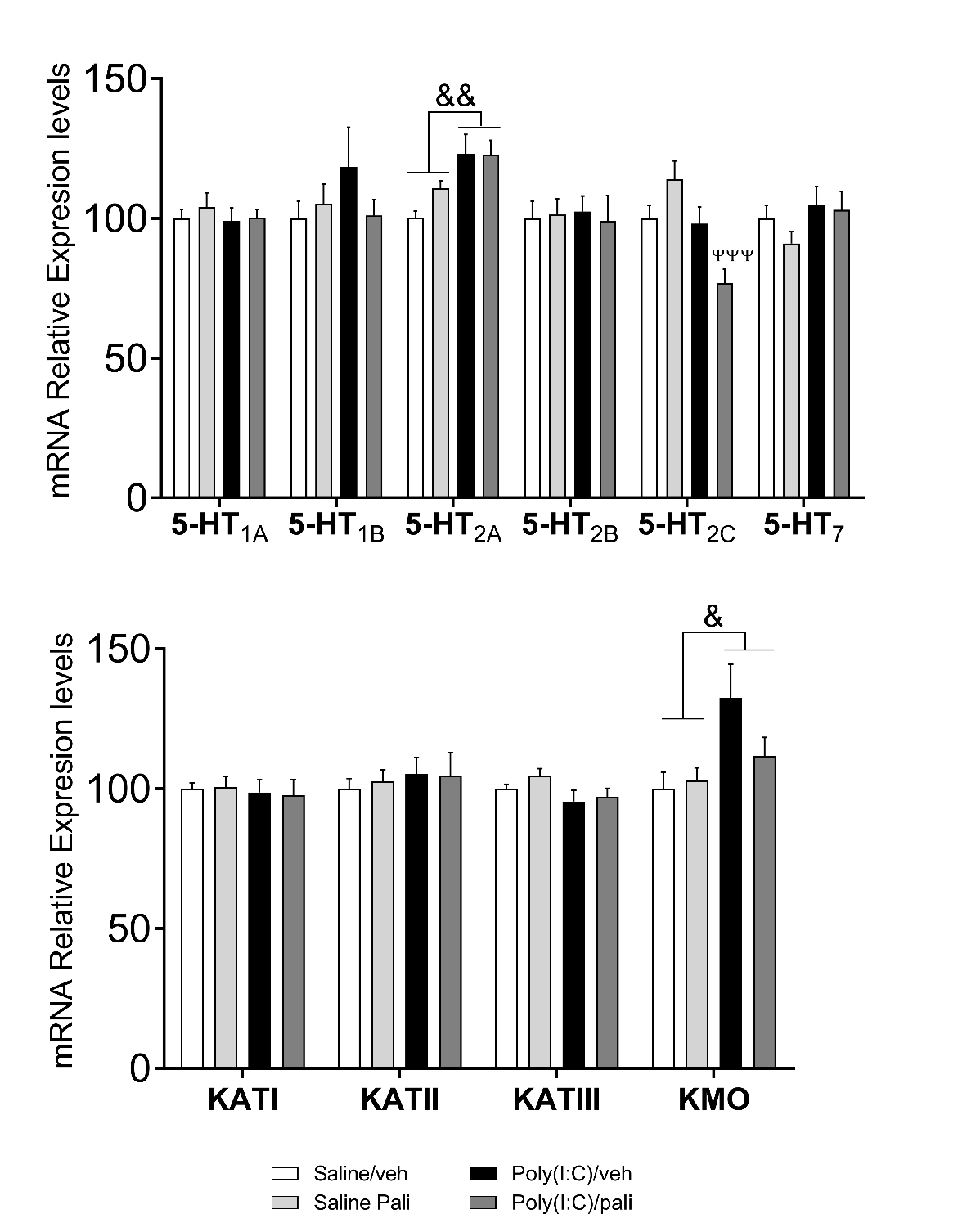


**Supplementary Figure 2.** Paliperidone effect on mRNA expression levels of serotonin 5-HT receptors and enzymes of kynurenine pathway in animals exposed to MIA. Bars represent means±SEM. The number of experiments in all parameters analyzed was between 7-9 animals. Two-way ANOVA followed by Bonferroni post-hoc test. ^&^p< 0.05, ^&&^p<0.01 for Poly(I:C) factor. ^ΨΨΨ^p<0.001 vs saline/pali group (Bonferroni *post* *hoc* test after significant interaction) pali: paliperidone; veh: vehicle.

## Supplementary Tables

**Supplementary Table S1**. Specific primers for RT-PCR assays

|  | **Forward primers (5'-3')** | **Reverse primers (5'-3')** |
| --- | --- | --- |
| **5-HT_1A_** | TACTCCACTTTCGGCGCTTT | GGCTGACCATTCAGGCTCTT |
| **5-HT_1B_** | AGTCCTGCTGGTTGCTTTGT | ATCAGGTAGTTAGCCGGGGT |
| **5-HT_2A_** | TCTCACCATTGCGGGAAACA | GCCACCGGTACCCATACAG |
| **5-HT_2B_** | CCATTTCCCTGGACCGCTAT | TGGGATGGCGATGCCTATTG |
| **5-HT_2C_** | GCTGGACCGGTATGTAGCAA | CTGAAACTCCTATTGATATTGCCCA |
| **5-HT_7_** | GCGGTCATGCCTTTCGTTAG | CCCAAGGTACCTGTCGATGC |
| **KATI** | TCTGGGAGGCATGTTCAGGA | TTTGGACATGGTGAGAGACTGG |
| **KATII** | TCCCAAAGCTGGCATGTTTC | CTGGAAGGCTGTATCCATCTGT |
| **KATIII** | GCACTTCTGCCAAAATGGCT | TTCACCACAGAAGGATCCGC |
| **KMO** | GACTGCCGTGGAGTCCTATG | GGAACCTTGTCAGGTCCGAG |
| **Tubulin** | CCCTCGCCATGGTAAATACAT | ACTGGATGGTACGCTTGGTCT |
| **GAPDH** | TGCACCACCAACTGCTTAGC | GGCATGGACTGTGGTCATGAG |

**Supplementary Table S2.** Results of the two-way ANOVA analyses of mRNA expression.

| Parameter | Poly(I:C) | Treatment | Interaction |
| --- | --- | --- | --- |
| 5-HT_1A_ | F_(1,29)_=0.32; p=0.571 | F_(1,29)_=3.83; p=0.540 | F_(1,29)_= 0.12; p=0.727 |
| 5-HT_1B_ | F_(1,29)_=0.55; p=0.460 | F_(1,29)_=0.40; p=0.531 | F_(1,29)_=1.42; p=0.242 |
| 5-HT_2A_ | **F_(1,29)_=13.07; p=0.001** | F_(1,29)_=1.15; p=0.290 | F_(1,29)_=1.22; p=0.278 |
| 5-HT_2B_ | F_(1,29)_=0.008; p=0.992 | F_(1,29)_=0.02; p=0.873 | F_(1,29)_=0.12; p=0.722 |
| 5-HT_2C_ | **F_(1,26)_=12.12; p=0.001** | F_(1,26)_=0.40; p=0.530 | **F_(1,26)_=9.92; p=0.004** |
| 5-HT_7_ | F_(1,29)_=2.24; p=0.144 | F_(1,29)_=0.92; p=0.343 | F_(1,29)_=0.38; p=0.539 |
| KATI | F_(1,29)_=0.23; p=0.630 | F_(1,29)_=0.002; p=0.965 | F_(1,29)_=0.03; p=0.863 |
| KATII | F_(1,29)_=0.38; p=0.538 | F_(1,29)_=0.03; p=0.862 | F_(1,29)_=0.07; p=0.780 |
| KATIII | F_(1,29)_=4.17; p=0.050 | F_(1,29)_=1.10; p=0.302 | F_(1,29)_=0.25; p=0.616 |
| KMO | **F_(1,29)_=6.28; p=0.018** | F_(1,29)_=1.16; p=0.288 | F_(1,29)_=2.08; p=0.159 |

Two-way ANOVA analyses (F, df and p values) for mRNAs determinations. Bold: statistically significant values.
